# Measurement of Head Circumference Using a Smartphone: Feasibility Cohort Study

**DOI:** 10.2196/54194

**Published:** 2024-02-14

**Authors:** Stefan Yordanov, Kalsoom Akhter, Jye Quan Teh, Jawad Naushahi, Ibrahim Jalloh

**Affiliations:** 1 Academic Division of Neurosurgery Addenbrooke’s Hospital, Cambridge University Hospitals NHS Foundation Trust University of Cambridge Cambridge United Kingdom

**Keywords:** head circumference, HC, hydrocephalus, neurosurgery, pediatric neurosurgery, paediatric neurosurgery, neurology, neuro, neurosurgeon, neurologist, mobile health, mHealth, app, apps, application, applications, digital health, smartphone, smartphones, pediatric, pediatrics, paediatric, paediatrics, infant, infants, infancy, baby, babies, neonate, neonates, neonatal, toddler, toddlers, child, children

## Abstract

**Background:**

Accurate head circumference (HC) measurement is essential when assessing neonates and infants. Tape measure HC measurements are prone to errors, particularly when performed by parents/guardians, due to individual differences in head shape, hair style and texture, subject cooperation, and examiner techniques, including tape measure placement and tautness. There is, therefore, the need for a more reliable method.

**Objective:**

The primary objective of this study was to evaluate the validity, reliability, and consistency of HC app measurement compared to the current standard of practice, serving as a proof-of-concept for use by health care professionals.

**Methods:**

We recruited infants attending the neurosurgery clinic, and parents/guardians were approached and consented to participate in the study. Along with the standard head circumference measurement, measurements were taken with the head circumference app (HC app) developed in-house, and we also collected baseline medical history and characteristics. For the statistical analysis, we used RStudio (version 4.1.1). In summary, we analyzed covariance and intraclass correlation coefficient (ICC) to compare the measurement's within-rater and interrater reliability. The *F* test was used to analyze the variance between measurements and the Bland-Altman agreement, *t* test, and correlation coefficients were used to compare the tape measurement to the measures taken by the HC app. We also used nonvalidated questionnaires to explore parental or guardians’ experiences, assess their views on app utility, and collect feedback.

**Results:**

The total number of recruited patients was 37. Comparison between the app measurements and the measurements with a tape measure showed poor reliability (ICC=0.177) and wide within-app variations (ICC=0.341). The agreement between the measurements done by parents/guardians and the tape measurements done by the researcher was good (ICC=0.901). Parental/guardian feedback was overall very positive, with most of the parents/guardians reporting that the app was easy to use (n=31, 84%) and that they are happy to use the app in an unsupervised setting, provided that they are assured of the measurement quality.

**Conclusions:**

We developed this project as a proof-of-concept study, and as such, the app has shown great potential to be used both in a clinical setting and by parents/guardians in their own homes.

## Introduction

Accurate head circumference (HC) measurement is essential when assessing neonates and infants. HC outside the normal range may indicate a brain development disorder, hydrocephalus, or an intracranial mass lesion. The growth pattern of HC, determined from serial measurements, provides valuable clinical information; for example, changes in HC are used to help determine whether hydrocephalus needs treatment and is predictive of neurodevelopmental outcomes [1].

The importance of HC measurement is recognized worldwide. The World Health Organization advises HC measurements just after birth (although measurements taken within the first 24 hours can be unreliable due to moulding), at the 8-week check, and any time thereafter if there are concerns about the child’s head growth, weight gain, development, or general health. It is also advisable for HC measurement to be performed at any pediatric review in the first 2 years of life. An accurate HC measurement is essential when clinically evaluating a neonate or infant. It monitors slow or excessive growth, assesses the impact of illness and treatment, and identifies those at higher risk of neurodevelopmental disorders [2]. HC should be measured at extremes of body weight (either below the 0.4th centile or above the 99.6th centile) or if there is rapid weight gain [3].

HC is the widest circumference of the head measured using a tape measure. Typically, this is performed by health care workers. With the increasing use of telephone and remote review clinics as a substitute for face-to-face appointments, community measurement of HC is more frequently used to help guide clinical management in various clinical specialties, such as neurosurgery, neonatology, and pediatrics. Parents and caregivers can be taught to measure the HC using tape, but its accuracy and reliability are sometimes insufficient for making clinical decisions. Many caregivers need more confidence to perform HC measurements themselves because of the technical challenges in performing the measurement and the potentially severe implications of erroneous measurements. Often, HC measurements must be checked by a health care professional, necessitating additional health visitor home visits or trips to the general practitioner.

HC measured with a tape measure is also prone to errors due to individual differences in head shape, hairstyles and texture, patient cooperation, and examiner techniques, including tape measure placement and tautness [4,5]. Measuring HC with a tape measure is often challenging due to poor cooperation in infants, particularly in a health care setting. Neonates in the neonatal intensive care unit pose significant challenges for HC measurements due to the risks associated with handling or removing them from the incubator. Many neonates require daily HC measurements.

The challenges of performing HC measurement with a tape measure, both in the hospital and the community, mean many missed opportunities to capture HC. Only 3% of infants presenting to Accident and Emergency had their head circumference measured within 1 year in an Australian hospital [6]. A study from a UK hospital found that HC measurement was performed sporadically in only 1 of 7 infants [7]. In summary, HC assessment is frequently missed due to difficulties measuring with a tape measure and the errors associated with this method. There is, therefore, an urgent need to improve our ability to monitor HC easily, accurately, and reliably, which would facilitate greater dependence on caregivers to perform this assessment independently.

We developed a smartphone app that measures HC using the smartphone camera and automated measurement to improve the accuracy and reliability of HC measurement. Our study’s main objective was to validate the accuracy of the HC app and prove its utility and feasibility when used by both health care workers and parents/guardians.

## Methods

### Patient Recruitment

Treating clinicians identified patients eligible for this study (infants <18 months of age) in a tertiary neurosurgical referral center and notified research team members with the consent of parents/guardians. Once identified, research team members approached patients’ parents/guardians to seek informed consent before their clinic appointment or at the bedside on the ward. An information sheet was used. If they chose to participate, we measured the HC during the clinic appointment or on the ward. Sample size estimation was calculated with 90% statistical power to detect a change of means by 1 and an SD of 1. This, along with 10% iteration, equaled 33 participants.

### App Development

To successfully develop the app, we built the back end and front end of the app separately. Languages used were Python and JavaScript, and the app was built in Expo [8] using React Native [9]. The app’s back end used an algorithm to recognize a reference object and provide a measurement according to the object. Technically, the algorithm consists of a couple of parts. One part includes the foreground and contour recognition code, which recognizes the oval object and then measures the pixel points around its contour. Another part of the algorithm detects the reference object and recognizes the scale in comparison. They both fuse using Python to measure according to the identified contour points of both objects and the introduced scale of the known object sizes, providing a measurement within the scale. Points were manually inputted to clarify the top, bottom, and outermost lateral edges of the baby’s head. This was introduced as an additional measure to align with the already recognized contour and provide a more accurate measurement.

The algorithm was tested to match an SD of 0.5 cm between measurements. This SD target was selected to mirror the accepted variation in tape measurements, aiming for consistency and reliability while minimizing bias and variability associated with traditional methods [10].

**Figure 1:**
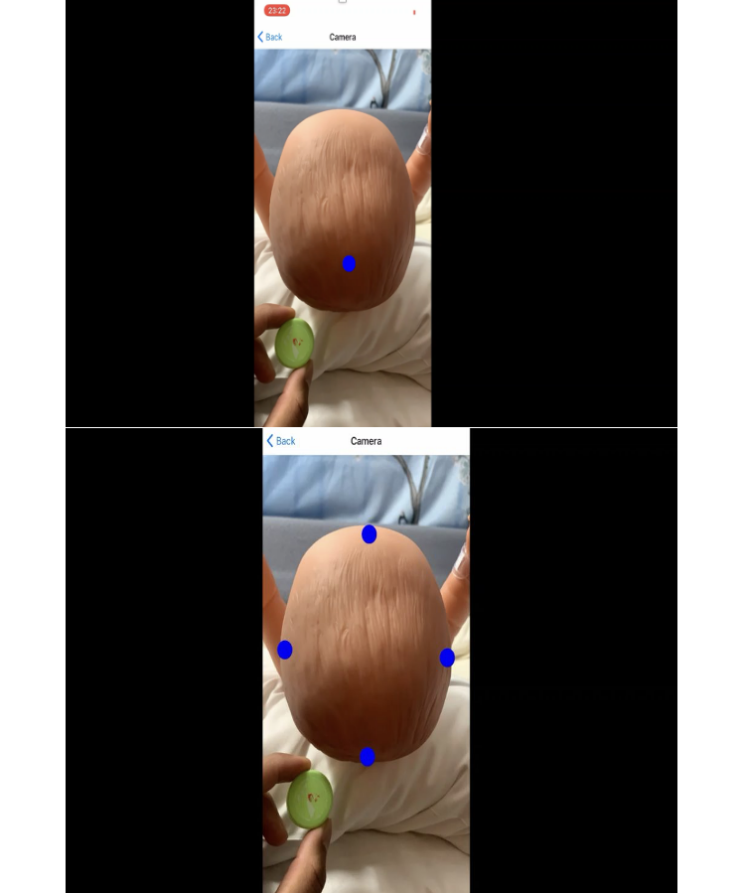
Head circumference app measurement sequence.

**Figure 2:**
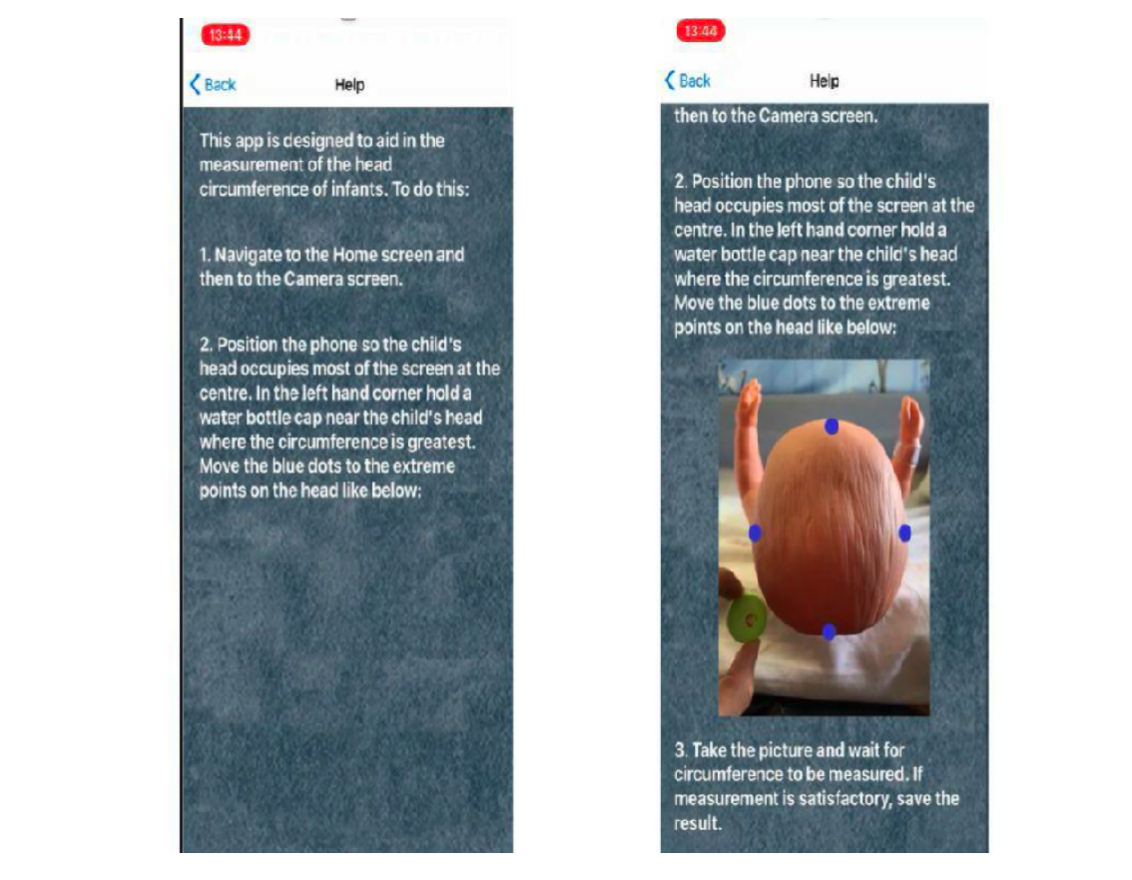
Parent/guardian instructions.

We used a phantom baby model to test the accuracy of our measurement model (Figure 1). Instructions for parents/guardians are shown in Figure 2.

### Ethical Considerations

Our regional research ethics committee (East of England-Cambridge East Research Ethics Committee) reviewed our study, and Health Regulatory Authority approval was obtained on June 6, 2022 (22/EE/0109). All parents/guardians of patients eligible to participate were approached with information about the study and given sufficient time for consideration of their participation. Informed consent in English was signed before any study-related procedures were undertaken. The data collected as part of the study were anonymized and deidentified. No patient identifiable data were collected as part of this study. Study participants and parents/guardians did not receive any compensation for their participation.

### Study Objectives

After the successful trial on a life-size baby model (3B Scientific W17001 Baby Care Model), we developed a trial protocol and a study. The rationale of our study was to validate the HC app in a clinical setting and to review its usability by parents/guardians in the community.

The primary objective was to assess the technical validity (ie, accuracy and precision) and user reliability (ie, consistency of measurements across different raters) of the HC app compared to standard tape measure methods. We also set multiple secondary objectives; these include evaluating user satisfaction with the HC app and comparing the reliability of HC measurements between health care professionals and parents/guardians using both tape and the app.

### Baseline and Study Data Collection

All participants had a medical history, clinical examination, and routine investigation details taken from their medical notes. The study phone did not retain any patient information. A nonvalidated questionnaire was used to capture parental or guardian feedback. Public and patient involvement feedback was sought in the design of the questionnaires for parental or guardian feedback. A complete list of questions can be found in Multimedia Appendix 1. HC measurements, as part of the study, were performed using a tape measure along the widest circumference of the baby’s head. Parents/guardians were instructed by a health care professional competent in HC measurement before the measurements were taken. They were also supervised during the procedure by a health care professional who was part of the study team. A specific set of instructions for using the app was prepared to be shown to the parents before they attempted to use the application. The instructions were developed with the help of a patient and a public representative group during the study development phase.

### Statistical Analysis

In this study, we introduce a novel measurement method, which requires comprehensive evaluation to ascertain its properties. Reliability, validity, and reproducibility are key criteria in measurement science. We adopt the COSMIN (COnsensus-based Standards for the selection of health Measurement INstruments) initiative standards, a widely recognized authority in clinical measurement quality [11] (Figure 2), to guide our assessment. In recent years, COSMIN has become a well-recognized organization [12], and consensus definitions can convey a unified message. COSMIN has also been dedicated to improving clinical measurement, including creating guidance and resources for measurement quality. That is why we decided to be guided by their definitions in exploring our novel measurement method’s reliability, consistency, and validity.

Guided by the COSMIN initiative, reliability is defined as the degree to which the measurement is free from measurement errors and includes a subsection of internal consistency (the degree of interrelatedness), reliability (the proportion of total variance due to true variance), and measurement error (systemic random error) [11,13]. To address this domain, we analyzed covariance, intraclass correlation coefficient (ICC), and Cohen Kappa and generated the interrater and within-rater reliability and variance. The ICC is between 0 and 1, where values below 0.5 indicate poor reliability, those between 0.5 and 0.75 indicate moderate reliability, those between 0.75 and 0.9 indicate good reliability, and any value above 0.9 indicates excellent reliability [14].

The COSMIN initiative also defines validity as the degree to which a measurement instrument measures the construct and its purpose to measure. The main subsections of this include content validity, criterion validity, and construct validity. Content validity ensures that the content of a measuring tool adequately reflects the given facts. Construct validity is defined as the degree to which the scores of a measurement instrument are consistent and includes structural validity, which is the degree of reflection of dimensionality, and hypotheses testing, which is synonymous with construct validity. Criterion validity shows how adequate the measurement is based on a “gold standard” [11,13]. For comparisons of agreements between the measures in this study, we used the correlation coefficients and the Bland-Altman method. The Bland-Altman plot analysis is a simple way to evaluate a bias between the mean differences and estimate an agreement interval. This interval encompasses 95% of the differences of the second method compared to the first one [15].

For the statistical analysis, we used RStudio (version 4.1.1). In summary, we analyzed covariance and ICC to compare the measurement’s within-rater and interrater reliability. The *F* test was used to analyze the variance between measurements and the Bland-Altman agreement, *t* test, and correlation coefficients were used to compare the tape measurement to the measures taken by the HC app.

## Results

### Demographics

We recruited 37 patients (23 male and 14 female), with a median age of 9 (IQR 4-16) months.

**Figure 3:**
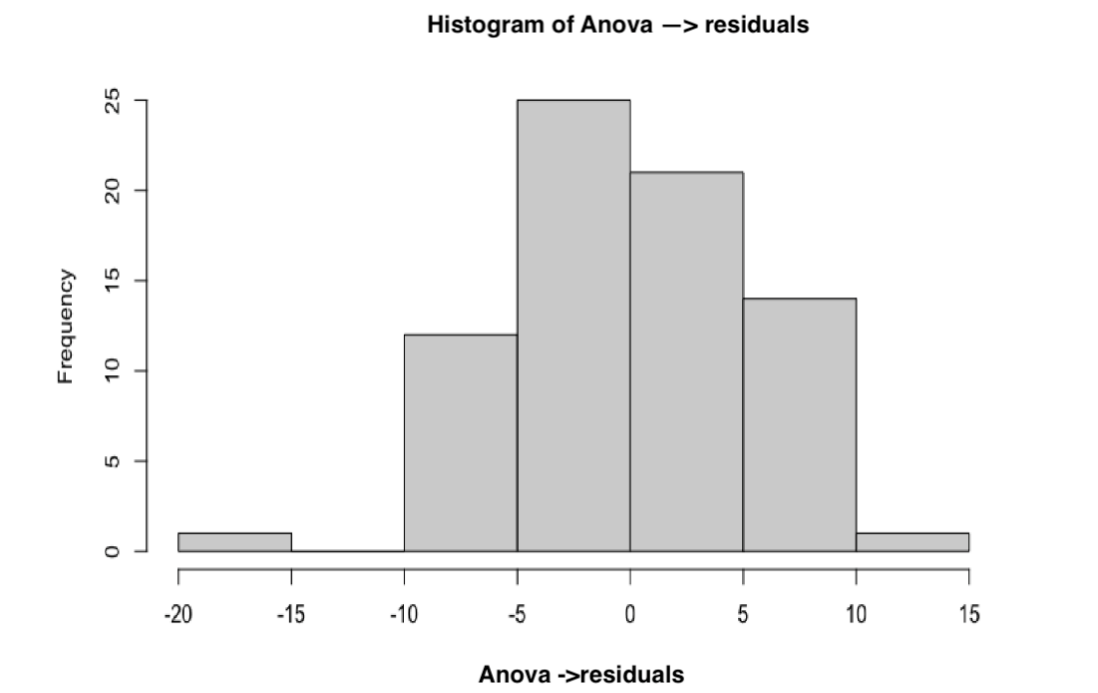
Histogram of residuals and assessments (ANOVA).

Normal means distribution was indicated visually with a histogram (Figure 3); however, the Shapiro-Wilk normality test with a *W*=0.777 and *P*<.001 showed a deviation from the normal distribution of means.

### Comparison of HC App Measurements Versus Tape Measurements

A 2-sample *t* test was used to compare the mean values of all the tape measurements with HC app–based measurements. There was a statistically significant difference between the measurements (*P*<.001). The variance in the population means was found to be equal (*F*_5,30_=0.92134; *P*=.81).

**Figure 4:**
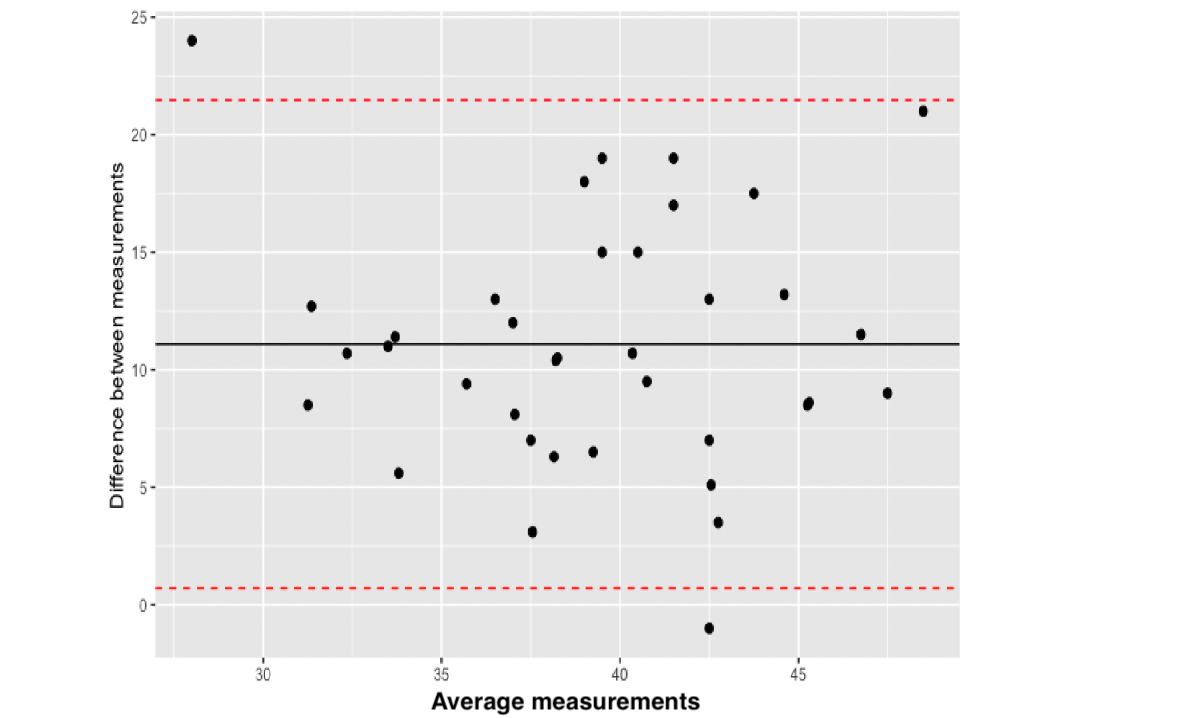
Bland-Altman plot; limits of agreement and variability assessment between app measurements and tape measurements.

We then calculated the limits of agreement between the 2 measures using the Bland-Altman plot. The upper limits of agreement were determined to be within a range of 0.706 to 21.472, revealing significant variability in the data points (Figure 4). These were scattered across the field away from the 0 lines, indicating a weak agreement between the 2 methods. We then assessed the ICC for the measurements, which showed a value of 0.177, which is less than 0.5, indicating inferior reliability of the HC app measurements in comparison to the measurements taken by a tape measure.

### Comparison of Measurements Within the App (Interrater Reliability)

**Figure 5:**
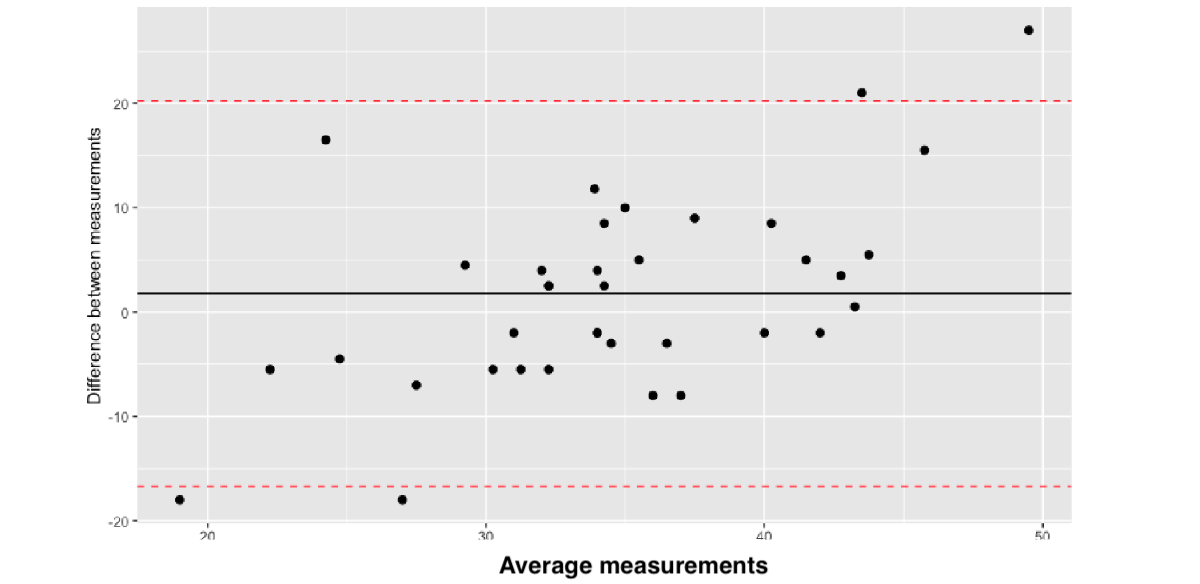
Bland-Altman plot; limits of agreement and variability assessment between different app measurements.

We compared the HC app’s performance between different raters (researchers and parents/guardians) by calculating the ICC, which yielded a value of 0.341. The limits of agreement, determined using Bland-Altman plots, were also high (lower limit –16.696 and upper limit 20.252), with a mean difference of 11.089 and with a significant scatter of the values. These results show poor agreement between the rater evaluations. (Figure 5). We then performed an *F* test to assess whether the variance between the 2 groups was equal. This resulted in *F*_35,71.4_=0.302 and *P*<.001, indicating a difference in the variance of the measurements between the groups. The same analysis was also performed comparing the consequential researcher measurements, showing similar results (*F*_3,33_=1.994; *P*=.04). The standard error of the mean (SEM) between the measurements done by parents/guardians and the researcher measurements was 0.979 and 0.921, respectively. Cohen Kappa was calculated at 0.435, showing a fair agreement between the measures.

### Comparison Between Parent/Guardian and Health Care Professional Tape Measurement

**Figure 6:**
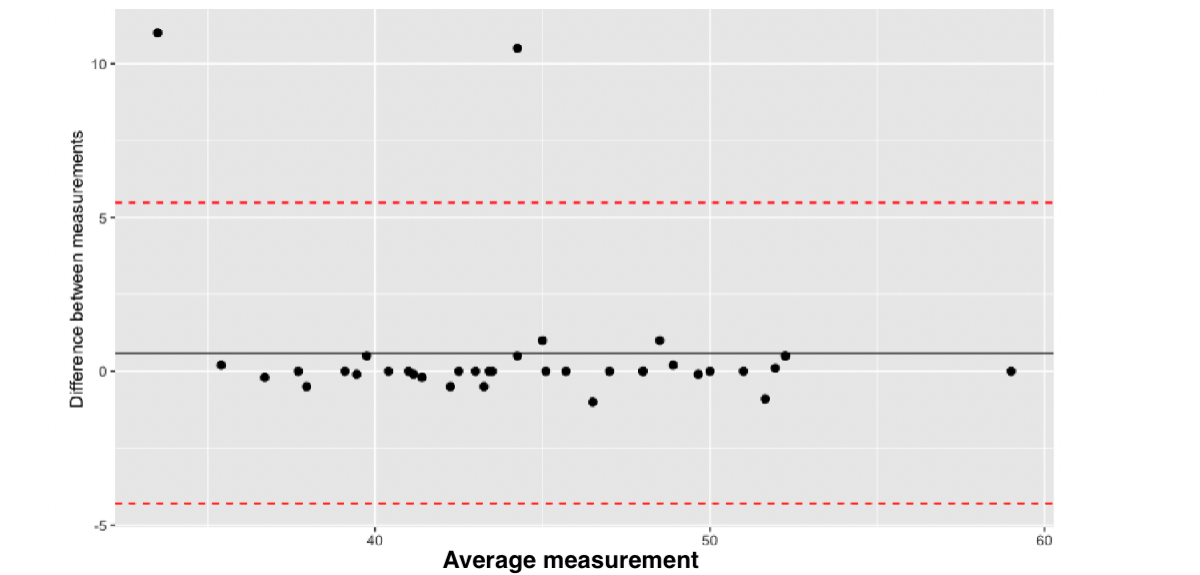
Bland-Altman plot; limits of agreement and variability assessment between parent/guardian and health care professional app measurements.

We also compared the interrater reliability between the measurements taken with a tape measure. The parents/guardians measured the HC under direct supervision and guidance. The ICC was 0.901, indicating good method reliability with very few lines above the 0 line in the plot and a mean difference of 0.5, comparable to other evidence in the literature [16]. The SEM for the medical professional measurements was 0.88, while the SEM for the parent/guardian measurements was 0.97. Cohen Kappa was set at 0.93, showing near-perfect agreement (Figure 6).

### User Feedback

The survey results showed favorable opinions from parents/guardians, with the majority of them being happy to use the HC app in a nonsupervised setting. Most parents/guardians (n=31, 84%) answered that the HC app was easy or extremely easy to use, and 33 (89%) responded that they were very confident in using the HC app after reviewing the instructions (Figures S1 and S2 in Multimedia Appendix 1). The app instructions, created using public and patient involvement feedback, were also valued by the parents (Figure S4 in Multimedia Appendix 1). Once again, the vast majority (n=32, 87%) were either satisfied (n=14) or very satisfied (n=18) with the HC app (Figure S3 in Multimedia Appendix 1). Free-text comments from several parents/guardians described that they found the concept of using the HC app appealing and were happy to be presented with the opportunity to use it. Most parents were also happy to use the app even at home if available (Figure S5 in Multimedia Appendix 1). Furthermore, they indicated that they would be very likely to use the HC app provided that the app’s measurements and reliability improve.

## Discussion

### Principal Findings

We developed a smartphone app that measures HC using the smartphone camera and automated measurement. Our HC app is less accurate in its current iteration than the standard tape measure. Interrater reliability using the app was poor, but there was no significant difference in the variability between the operators. Parents/guardians also valued the convenience of using the app and the ease of performing the measurements, highlighting the potential of this technology once modified to improve accuracy.

The idea for our HC app was conceived during the COVID-19 pandemic lockdown in 2020, when disruption to health care services significantly reduced face-to-face appointments between patients and health care providers. Parents/guardians can measure HC using tape, but this requires some teaching and is prone to errors. Many parents/guardians express anxiety about assessments typically carried out by health care professionals being delegated to them and used in making clinical decisions. We, therefore, recognized the opportunity to create an automated method for HC measurement that is simple and easy to use and eliminates the errors associated with tape measure placement and patient cooperation.

Smartphone (and wearable) technology plays an ever-increasing role in public health and health care delivery, increasing the ability of health care workers to monitor patients remotely and empowering patients to track their health care metrics. The potential of technology to benefit health care is recognized by the World Health Organization, which introduced the term “mHealth” [16] to denote “medical and public health practice supported by mobile devices, such as mobile phones, patient monitoring devices, personal digital assistants (PDAs), and other wireless devices” [16]. Mobile communication allows remote assessments with results that can be transferred distantly via mobile devices. In this landscape, we feel that replacing the tape measure with a smartphone equivalent is inevitable.

The current version of our HC app requires a reference object and manually placed input points to indicate the top, bottom, and lateral edges of the baby’s head. The high reproducibility of measurements taken by the same person indicates the ease and reliability of this method. The relatively poor accuracy of our HC app is likely due to confounders such as the photo’s angle and distance and the type of reference object used. Using a bright-colored reference object (eg, a bottle cap) gave us a more accurate reading. However, we recognize that this choice of reference object may also relate to some inaccuracies, making the app less user-friendly and contributing to the difficulties in obtaining the perfect conditions for the photo. Thus, we intend to continue to develop the HC app using newer smartphone technology, such as light detection and ranging (LIDAR), which will improve the accuracy of the measurements, eliminate the need for a reference object, and make the app more user-friendly. This technology is highly advanced, and mobile LIDAR is already incorporated into most modern phones. Since 2020, Apple Inc has introduced a LIDAR sensor in some iPhone and iPad models, namely iPhone 12 Pro, iPhone 12 Pro Max, iPhone 13 Pro, iPhone 13 Pro Max, iPad Pro 2020, and iPad Pro 2021 (as of March 2022) [17]. Dimension can easily be measured between 2 points in the LIDAR 3D point cloud, providing accurate measurements of various objects. This technology is already used in other fields [18,19]. Thus, we are confident that this technology can be reliably used to improve the measurement accuracy and usability of the app.

Feedback from parents/guardians about the HC app revealed that they are confident in using the HC app and value the ability to track HC themselves in the convenience of their homes. They appreciated the ease of performing the measurements and performing them without fear of waking a sleeping infant. Overall, we received positive feedback from both the public and patient panel groups we consulted in preparation for our proof-of-concept study as well as during the trial itself. Many parents gave both written and verbal feedback with ideas and suggestions for how to improve the app’s utility. Most parents suggested features they would like to see, which shows good community engagement, reassuring that the concept can easily reach the target auditorium. Most of the parents were very welcoming to the technological solution we proposed. They expressed their interest in using it in the privacy of their homes after improvements to the app. However, reassurance is needed that the measurements are accurate and reproducible, which is something to be aware of when the final version of the app is distributed. We will keep that in mind in the development of future studies following the development of the final app to increase the adoption in the community.

Importantly, our study confirms that parent/guardian measurements using a tape measure are reliable with an excellent correlation (ICC=901) and agreement (kappa=0.93) between health care professionals and parents/guardians. Although our findings are similar to those previously reported by Sullivan et al [4] and support empowering parents/guardians to perform these measurements, these measurements were done under the direct supervision of a health care professional with an additional helping hand during the measurements. Having this in mind, translating these results may provide false reassurance regarding the reliability of parental measurements done at home without supervision. The only thing we can do to manage erroneous measures is to provide sufficient training and guidance to parents/guardians. This does not eliminate errors, but it offers a certain degree of reassurance. The gold standard measure, nevertheless, will remain the one measured by a health care professional. In contrast, the HC app, once improved, has the potential to introduce a sustainable, uniform, and reproducible means of measurement, which can be applied consistently across settings, providing equal reliability.

### Limitations

To facilitate recruitment and combined measurements by both health care professionals and parents/guardians within a reasonable time frame, measurements were performed in the clinic rather than at home. A researcher supervised measurements; however, once instructions were given, no further help was provided, as our intention was for the HC app to be reliably used by parents/guardians independently at home. Our study design, therefore, only partially validates the HC app use in the home environment independent of health care professionals. We plan to explore this with future iterations of our HC app. Another important limitation to report is associated with the Shapiro-Wilk normality test. As the data set increases in size, the test can pick up very small variations, which can result in a higher likelihood of rejecting the null hypothesis [20].

### Conclusions

Our HC app has demonstrated proof-of-concept for parent/guardian HC measurement using smartphone technology. The feedback collected from parents/guardians confirmed that the technology is easy to use, giving them the confidence to perform the measurement independently. Overall, parents/guardians were interested in this technological solution and were eager to give both written and verbal feedback during the study. This, along with the clinical proof-of-concept, reassured us that the technology is feasible, prompting us to initiate plans to improve the versions of our HC app.

## Appendix

Multimedia Appendix 1 Questionnaire and additional statistics.

## Abbreviations

COSMIN: COnsensus-based Standards for the selection of health Measurement INstruments
HC: head circumference
ICC: intraclass correlation coefficient
LIDAR: light detection and ranging
PDA: personal digital assistant
SEM: standard error of the mean

## Data Availability

The data sets generated during this study are available from the corresponding author upon reasonable request and after approval from the sponsor.
