# Supplementary material for: Measurement of Head Circumference Using a Smartphone: Feasibility Cohort Study
Source: JMIR Form Res. 2024 Feb 14;8:e54194. doi: 10.2196/54194 (PMC10902771; doi:10.2196/54194)
Supplement: Multimedia Appendix 1 [file formative_v8i1e54194_app1.docx]

**Supplementary Material:**

**Parent Feedback Questionnaire:**Questionnaires.

1. ***How easy did you find the use of the app?***

- Extremely easy
- Easy
- Unsure
- Hard
- Extremely hard

1. ***How confident do you feel using the app?***

- Very Confident
- Confident
- Unsure
- Not confident

1. ***Would this app be preferable to having appointments to have the measurement taken?***

- Yes
- No

1. ***Are the app instructions clear and easy to use?***

- Yes
- No
- Comments………………………………………………………………………………………………..
  …………………………………………………………………………………………………………………

1. ***Are there any other features you would like to include?***
   …………………………………………………………………………………………………………………..

……………………………………………………………………………………………………………………**………….**

……………………………………………………………………………………………………………………………….
……………………………………………………………………………………………………………………………….

……………………………………………………………………………………………………………………………….
……………………………………………………………………………………………………………………………….

1. ***Are you satisfied with your experience of using the app?***

- Very Satisfied
- Satisfied
- Unsure
- Dissatisfied
- Very Dissatisfied

1. **Do you have any other comments regarding the app?**

……………………………………………………………………………………………………………………………….
……………………………………………………………………………………………………………………………….

……………………………………………………………………………………………………………………………….
……………………………………………………………………………………………………………………………….

……………………………………………………………………………………………………………………………….
……………………………………………………………………………………………………………………………….

1. **Would you use this app at home if available?**

- Yes
- No

***Thank you!***

**Figure S1. Answer distribution Question number 1 in the questionnaire**

**Figure S2. Answers were distributed to question number 2 in the questionnaire**

**Figure S3. Answer distribution of question Number 6.**

**Figure S4. Answer distribution to Question Number 4 in the questionnaire**

**Figure S5. Answer the distribution of question Number 8 from the questionnaire.**
